# Highlight: Andean Hummingbirds Reveal Distinct Evolutionary Pathways to High-Altitude Adaptation

**DOI:** 10.1093/gbe/evz126

**Published:** 2019-06-27

**Authors:** Casey McGrath

The transition from living at low elevations to high elevations, such as those found in the Andes, Himalayas, and Tibetan Plateau, comes with many challenges. Extremely high elevations are associated with colder temperatures, increased exposure to ultraviolet radiation, and a lack of oxygen, which is 40% less abundant above 4,000 m than at sea level. Organisms that live at these elevations have developed specific adaptations that allow them to cope with these difficulties. For example, genetic analyses of humans living in the Andes and on the Tibetan Plateau have revealed changes in specific genes that enable them to deal with limited oxygen environments. However, it remains unclear whether such changes are generally predictable across different species or populations, or whether there is some flexibility in what changes occur. In a new article in *Genome Biology and Evolution* (Lim et al. 2019), a group of researchers from Stony Brook University, the University of New Mexico, and the Swiss Federal Research Institute set out to answer this question.

According to the study’s first author, Marisa Lim, a PhD candidate at Stony Brook University at the time of the research, “The biology and evolutionary history of hummingbirds presented the perfect system to study parallel evolution, as Andean hummingbirds occur at very high elevations and high-altitude species evolved independently across the hummingbird family.” By studying a group of 12 Andean hummingbird species that represent several independent transitions to higher altitudes, the researchers hoped to determine whether changes that confer benefits at high elevations occur in similar amino acids, proteins, or biochemical pathways across species.

The researchers sequenced almost 1,000 genes from each of the 12 species and identified genes and amino acid sites that were under positive selection and were shared across high- or low-elevation hummingbird species. While some changes at particular amino acid sites or in particular genes were shared across species, broadly speaking, the researchers found greater similarity in the overall biochemical pathways and biological functions associated with adaptation to high altitudes. For example, positively selected genes were often involved in pathways or functions related to cellular respiration, metabolism, or mitochondrial biogenesis and translation, with additional genes involved in cell death and immune function.

This finding, that “Andean hummingbirds have converged on the high-altitude phenotype through the evolution of different genetic mechanisms,” is the study’s most exciting result according to Dr Lim. She believes this indicates that there is “predictability in the evolutionary machinery, primarily across biochemical pathways” and suggests that there is some functional overlap among genes that allows for flexibility in how adaptations evolve.

David Hillis, a professor at the University of Texas at Austin who was not involved in the study and who has studied high-elevation adaptation in Tibetan frogs and lizards (Sun et al. 2018), agrees, stating that the hummingbird study “suggests that even among relatively closely related species that exhibit parallel shifts to high elevations, the details of the molecular adaptations are largely different.” Dr Hillis notes that the study also suggests that parallel evolution in function is unlikely to confound phylogenetic studies and fits experimental results from laboratory evolution of viruses.

Dr Lim anticipates future challenges associated with a lack of knowledge about gene function, particularly in nonmodel organisms, which may limit the ability to “discern true signatures of positive natural selection from statistical and biochemical noise.” While she notes that their results “support and are supported by our current knowledge of genetic adaptation to high-altitude conditions,” she acknowledges that the story does not end here and hopes that future research can build upon the study’s findings. Building upon this study, she is now investigating the adaptive evolution of high- and low-altitude populations using population genomic methods. Dr Lim recently became a bioinformatics postdoctoral fellow with the Wildlife Conservation Society at the Bronx Zoo, where she develops user-friendly bioinformatics pipelines for wildlife conservation projects.
